# Federated learning for cardiovascular disease prediction: a systematic review of clinical applications, validation, and translation readiness

**DOI:** 10.3389/fcvm.2026.1831342

**Published:** 2026-06-08

**Authors:** Jie Li, Wei Xiang, Dandan Shang, Shujuan Li, Qin Li

**Affiliations:** 1 School of Medical Science and Engineering, Beijing Institute of Technology, Beijing, China; 2 School of Medical Imaging, Hebei Medical University, Shijiazhuang, China; 3 School of Basic Medicine, Hebei Medical University, Shijiazhuang, China; 4 Department of Neurology, Fuwai Hospital, National Center for Cardiovascular Diseases, Beijing, China

**Keywords:** cardiovascular disease prediction, clinical decision support, electronic health records, federated learning, machine learning, model validation, multi-center studies, privacy-preserving

## Abstract

**Background:**

Data silos and privacy constraints limit the centralized development of machine learning models for cardiovascular disease. Federated learning enables multi-institutional training without sharing raw patient records, but the evidence base in cardiology and deployment-grade evaluation remains uneven.

**Objective:**

To synthesize how federated learning has been implemented for cardiovascular disease prediction and to identify factors that determine clinical translation readiness, including heterogeneity handling, validation quality, privacy and security safeguards, and operational feasibility.

**Methods:**

A systematic literature review was conducted in PubMed, Web of Science, and IEEE Xplore, with supplementary searches in arXiv and Google Scholar, covering studies published from January 2022 through December 2025. Studies applying federated learning to clinically meaningful cardiovascular disease prediction tasks were included. We extracted data on clinical tasks, modalities, federation types, training strategies, evaluation designs, and deployment considerations, and synthesized the findings qualitatively.

**Results:**

Twenty-two studies were included, spanning early screening, clinical diagnosis, prognostic evaluation, and emerging treatment-related decision-support applications. Modalities included electronic health records, electrocardiograms, phonocardiograms, echocardiography, cardiac imaging, and wearable data. Most studies used horizontal federated learning with FedAvg baselines, with variants targeting non-IID heterogeneity, personalization, and efficiency. Some studies reported that FL performance approached centralized training or exceeded local baselines. However, the evidence base was predominantly retrospective and frequently relied on public datasets or simulated client splits, while reporting of held-out-site validation, calibration, subgroup performance, privacy safeguards, robustness, and system costs was inconsistent.

**Conclusion:**

Federated learning is a promising paradigm for privacy-preserving, multi-institutional cardiovascular disease prediction, yet the evidence remains mainly retrospective, with heterogeneous validation and limited deployment-grade reporting. This review synthesizes task, modality, federation design, and operational constraints, and highlights adoption priorities: held-out-site and external validation with calibration and subgroup fairness monitoring, communication-efficient personalization under drift, auditable privacy and security safeguards, and clinical-grade MLOps for monitoring, rollback, and continual updating.

## 1 Introduction

Cardiovascular disease (CVD) is one of the top causes of death worldwide, posing significant threats to human health. The prevalence of CVD continues to rise. The estimated number of current patients in China has reached approximately 330 million (1). In addition to long-term prevention and chronic management, time-sensitive deteriorations also contribute substantially to adverse outcomes.

With rapid advances in artificial intelligence (AI) for healthcare, disease prediction has become a central application area. Predictive models evaluate disease risk and progression, estimate prognosis, and personalize treatment, thereby enhancing clinical decision-making and informing public-health planning (2). In particular, early identification of high-risk populations, precise treatment planning, real-time monitoring of disease progression, and accurate prognosis evaluation are critical for reducing mortality and improving outcomes in major diseases such as CVD (3). Deep learning (DL) has demonstrated significant potential by extracting complex structural features from extensive clinical data to establish effective predictive models (4). Recent work has shown several useful examples. EchoNet-Dynamic can estimate beat-by-beat cardiac function from echocardiography (Echo) (5). Another study used an interpretable electrocardiogram (ECG)-based model for myocardial infarction classification by combining DenseNet and Grad-CAM (6). Other researchers also used instance segmentation to quantify cardiomyocyte hypertrophy for inhibitor screening (7). These advances highlight DL as a robust technological enabler for CVD risk assessment and early detection.

Despite these advances, most DL models rely on centralized learning paradigms that aggregate patient data from multiple institutions into a unified platform for training. Because this process handles sensitive patient data and must comply with strict legal and regulatory requirements, institutions rarely share data across sites, which entrenches persistent data silos (8). Even after anonymization, patient records may remain vulnerable to re-identification, creating residual risks of privacy leakage. As a result, centralized models often cannot fully leverage multi-center data and may show limited generalizability across institutions (9). Federated learning (FL) (10) offers a distributed way for institutions to train models together without sharing raw data. Studies have shown that models trained with FL can achieve performance comparable to centralized training and outperform models trained on data from a single institution (11). However, evidence and reporting remain inconsistent across studies, and practical challenges persist, including non-independent and identically distributed (non-IID) client data, communication and systems costs, privacy and security threats, and unresolved issues in interpretability and personalization.

To address this gap, we present a systematic review of FL-specific evidence for CVD prediction, focusing on studies that explicitly implemented FL rather than adjacent privacy-preserving distributed-learning paradigms. We examine the current evidence with a focus on clinical translation. We organize the literature by clinical task, federation design, evaluation strategy, and deployment considerations. This review makes three main contributions. First, we map how FL has been applied across major cardiovascular prediction tasks, from early screening to treatment-related decision support. Second, we summarize evaluation practices that shape multi-center generalizability, with emphasis on held-out-site and external validation, calibration, and subgroup performance reporting. Third, we synthesize underreported evidence on clinical deployment, including privacy and security safeguards, communication and system costs, and monitoring and update practices for clinical-grade systems. Together, these perspectives help clarify current priorities for clinical translation and highlight reporting gaps in the existing literature.

## 2 Federated learning

### 2.1 Overview of federated learning

FL lets multiple medical institutions train a shared model together. Each institution maintains patient-level data on site. Each institution updates the model locally. The institutions share only model updates, such as parameters or gradients. This process can reduce privacy and compliance risks compared with centralized data pooling (12). According to the distribution characteristics of data sources, FL can be categorized into three types (10):

Horizontal Federated Learning (HFL): samples across multiple datasets share the same feature space, but different samples reside in different datasets (10). It suits medical institutions with similar features and minimal cohort overlap, enabling collaborative training by aggregating local model updates while keeping raw data on site.

Vertical Federated Learning (VFL): Datasets share the same samples but differ in their features (10). It suits medical institutions with substantial patient overlap but complementary feature sets, enabling joint modeling via privacy-preserving entity matching and encrypted collaboration without sharing raw records.

Federated Transfer Learning (FTL): When two institutions have little overlap in patient cohorts or feature spaces, FTL can address data scarcity and label sparsity (10). It transfers knowledge across sites and modalities to support collaborative learning without sharing raw data, improving generalization when sample sizes are limited.

### 2.2 Research workflow of federated learning in disease prediction

**Figure 1:**
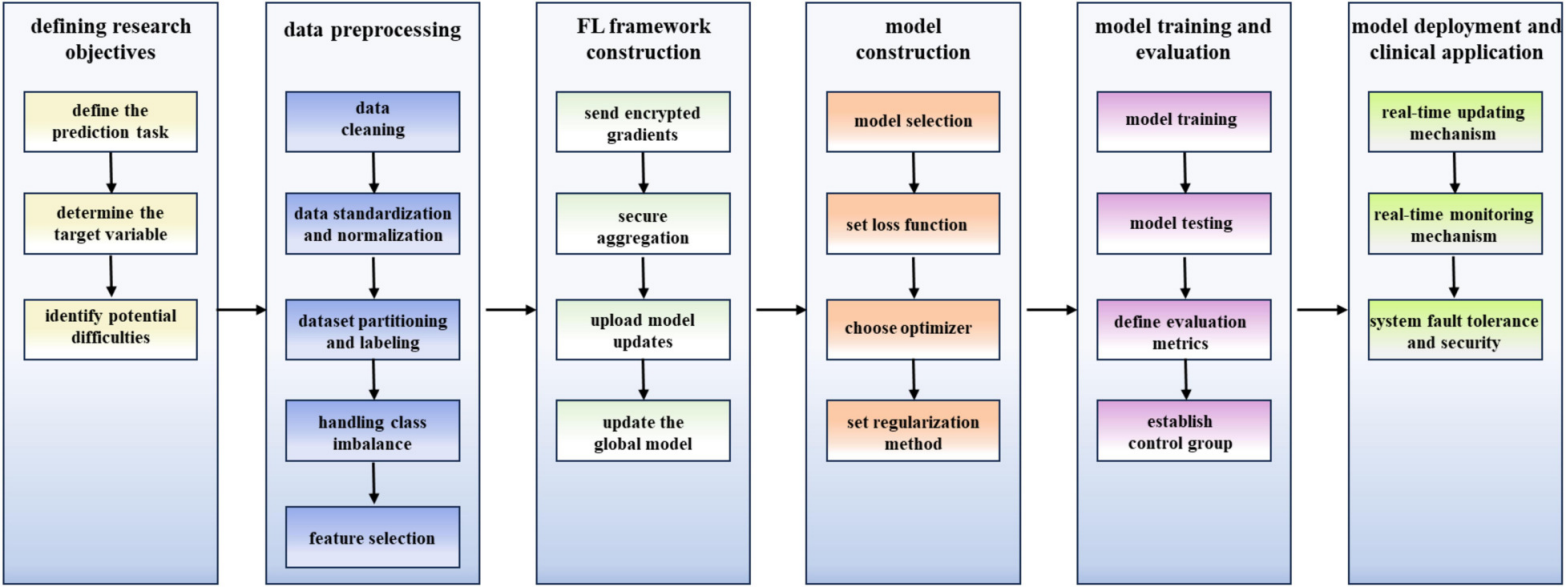
Research workflow of FL in disease prediction.

Figure 1 provides the organizing scaffold for this review by outlining the end-to-end workflow of federated learning for disease prediction, from objective definition and data preparation to federation design, model specification, training, evaluation, and deployment. We used this workflow to chart each included study with a standardized extraction form and to populate the structured summaries in Tables 1–4.

**Table 1:** Overview of FL applications in early screening of CVDs.

Ref	Clinical Task	Data source	Data Type	Client definition	Method	Validation design	Results
Alasmari et al. (13)	Early multimodal CVDs screening	Public datasets	CMR, ECG, EHRs, nutrition metadata	Simulated clients+server	FL Framework: FedAvg Core Model: SGD-optimized DNN Strategy: attention-based multimodal feature fusion	Internal split; Within-site; Centralized vs. FL, FL variants, Multimodal vs. Unimodal	FedAvg multimodal model: Accuracy 99.12%; federated unimodal ECG: Accuracy 97.45%
Qiu et al. (14)	Normal vs. abnormal heart sound classification	PhysioNet/CinC 2016 heart sound databases (a–f)	PCG-derived images	4 simulated sites (DC–DF) + server	FL Framework: FedAvg Core Model: CNN Strategy: 10% globally shared data for initialization (Non-IID mitigation)	Fixed validation/test split (DA/DB); Cross-dataset; FL variants	Non-IID setting with shared-data initialization: Accuracy 65.4%, Sensitivity 59.2%, Specificity 65.9%; without shared-data initialization: Accuracy 62.0%
Qiu et al. (15)	Abnormal heart sound detection	PhysioNet/CinC 2016 heart sound databases (a–f)	ComParE features	2-party VFL (guest with labels; host without labels)	FL Framework: VFL Core Model: Vertical-SecureBoost Strategy: Naive PU with label masking (20% positives labeled)	Fixed train/validation/test split; Cross-database; FL variants	At PU1 proportion 0.3: Accuracy 84.36%, Unweighted average recall 84.33%, Unweighted F1 84.35%
Alreshidi et al. (16)	Atrial fibrillation prediction	Public dataset/PhysioNet (challenge-2017)	ECG	Simulated clients+server	FL Framework: Fed-CL (FedAvg) Core Model: MCNN-LSTM Strategy: feature extraction+SMOTE	Internal split; Within-site; Centralized ML/DL vs. FL (Fed-CL)	After feature extraction with SMOTE: Accuracy 95.25%, Precision 96.54%, Recall 97.32%, F1-score 96.93%

Validation design is summarized using three label dimensions used consistently in Tables 1–4: validation scheme, test scope, and comparator. Performance metrics are reported as presented in the original studies.

CMR, cardiac magnetic resonance; CNN, convolutional neural network; DL, deep learning; DNN, deep neural network; ECG, electrocardiogram; EHR, electronic health record; FedAvg, federated averaging; FL, federated learning; LSTM, long short-term memory; MCNN, multi-scale convolutional neural network; ML, machine learning; non-IID, non-independent and identically distributed; PCG, phonocardiogram; PU, positive-unlabeled; SGD, stochastic gradient descent; SMOTE, synthetic minority over-sampling technique; VFL, vertical federated learning.

**Table 2:** Overview of FL applications in the diagnosis of CVDs.

Ref	Clinical Task	Data source	Data Type	Client definition	Method	Validation design	Results
Morbach et al. (17)	Automated echocardiographic measurement refinement	STAAB cohort; WASE study	Echo	2 sites+server	FL Framework: sequential model transfer Core Model: cascaded CNN detectors Strategy: cross-site re-training to reduce inter-reader variability	Internal split; Within-site; Original vs. re-trained detector vs. human	Original vs. Human: LVDd mean difference −1.1 mm Re-trained vs. Human: LVDd mean difference 0.2 mm; B < A in 66% (LVDd), 69% (E)
Zhang et al. (18)	ECG classification and Echo segmentation	FedCVD	12-lead ECG; Echo	Fed-ECG: 4 clients; Fed-ECHO: 3 clients+server.	FL Framework: FedAvg Core Model: ResNet1d-34 for ECG and U-net for Echo Strategy: Natural partitioning; Supervised-only masking for incomplete labels	Internal split; Cross-site (global testing); FL variants	ECG (GLOBAL): FedAvg micro F1-score 67.9 ± 3.8; mean average precision 50.8 ± 0.4; FedProx micro F1-score 68.8 ± 2.6; mean average precision 52.3 ± 0.9; Echo (GLOBAL): FedAvg Dice coefficient 50.2 ± 5.3; FedInit Dice coefficient 79.5 ± 0.5
Tölle et al. (19)	TAVI planning: CT landmark detection and calcification segmentation	Real-world multi-center cohort from 8 hospitals	Cardiac CT	8 hospital clients+server; mixed labeled/unlabeled data	FL Framework: FedAvg Core Model: 3D-ResUNet teachers; SWIN-UNETR student Strategy: Two-step semi-supervised federated knowledge distillation	Held-out-site validation; Cross-site; Local vs. FL vs. variants	Semi-supervised, labeled+unlabeled clients Dice coefficient: Local UNet 0.644 ± 0.290; FedSWIN-UNETR 0.692 ± 0.232; FedKD-SWIN-UNETR 0.670 ± 0.231
Linardos et al. (20)	HCM diagnosis from CMR	M&Ms and ACDC datasets	CMR	4 simulated centers+server	FL Framework: FedAvg Core Model: 3D-ResNet18 CNN Strategy: Equal-voting aggregation (FL-EV; uniform client weights)	Cross-validation; Cross-site (LCO-CV), Total+site-level; FL vs. FL variants	Total: FL AUC 0.731 ± 0.009; FL-EV AUC 0.768 ± 0.003; Sagrada Familia: FL AUC 0.807 ± 0.003; FL-EV AUC 0.854 ± 0.005
Goto et al. (21)	HCM detection using ECG and Echo	Real-world cohort from BWH, MGH, UCSF, Keio University Hospital	12-lead ECG; Echo	3 sites+server(training)	FL Framework: FedAvg Core Model: CNN ECG model; 3D-CNN Echo model Strategy: Stepwise screening, ECG then Echo model	Internal split+External validation; External cohort; Single-site vs. FL, Human experts vs. FL	External validation (site-wise): FL AUC 0.90–0.96; single-site AUC 0.79–0.82; External cohort(0.5% prevalence), stepwise screening: Stepwise FL Sensitivity 0.84; Human experts Sensitivity 0.59
Yaqoob et al. (22)	Binary risk prediction of heart disease	Cleveland; Statlog; Hungary; Long Beach; Switzerland	Tabular clinical features	5 HSP clients+1 server	FL Framework: FedMA Core Model: RB-SVM Strategy: MABC feature selection	Internal split; Within-site; FL variants	Global FL: Accuracy 93.8%, Precision 94.2%, Sensitivity 96.6%, Specificity 81.8%; Improved communication efficiency by reducing rounds by ∼37.8%
Khan et al. (23)	Diagnosis of CVD	UCI (DS1); Switzerland (DS2)	Tabular clinical features	5 simulated clients+server	FL Framework: Asynchronous FL (AFLCP) Core Model: DNN Strategy: temporally weighted aggregation	Internal split; Within-site; FL variants	DS2: Async-FL (10 nodes): Accuracy 0.899, F1-score 0.874; DS2: Sync-FL (10 nodes): Accuracy 0.863; F1-score 0.849; DS1: Async-FL (6 nodes): Accuracy 0.891; F1-score 0.887
Houssein et al. (24)	Heart disease presence classification	UCI & IEEE heart disease datasets	Tabular clinical features	25 clients+server	FL Framework: HFL Core Model: CNN Strategy: FedImpPSO; gBest and gWorst score-based client selection	Cross-validation; Within-site; FL variants	FedImpPSO: IEEE: Accuracy 91.18%, Precision 95.45%, F1-score 93.50%; UCI: Accuracy 92%, Precision 91.84%, F1-score 91.84%

Label definitions for validation design are the same as in Table 1.

AUC, area under the curve; CMR, cardiac magnetic resonance; CNN, convolutional neural network; CT, computed tomography; DNN, deep neural network; ECG, electrocardiogram; Echo, echocardiography; FedAvg, federated averaging; FedKD, federated knowledge distillation; FedMA, federated matched averaging; FedProx, federated proximal optimization; FL-EV, federated learning with equal-voting aggregation; HCM, hypertrophic cardiomyopathy; HFL, horizontal federated learning; HSP, health service provider; KD, knowledge distillation; LCO-CV, leave-center-out cross-validation; LVDd, left ventricular end-diastolic diameter; MABC, modified artificial bee colony; PSO, particle swarm optimization; SVM, support vector machine; TAVI, transcatheter aortic valve implantation.

**Table 3:** Overview of FL applications in prognosis prediction of CVDs.

Ref	Clinical Task	Data source	Data Type	Client definition	Method	Validation design	Results
Archetti et al. (25)	Prognosis prediction in heart failure patients	Lombardy Heart Failure Dataset	EHR	23 medical institutes+server	FL Framework: FedSurF++ Core Model: Random Survival Forest Strategy: tree sampling by Concordance Index	Internal split; Within-site; Local vs. Centralized vs. FL	Local AUC 61.7 ± 0.9; Global AUC 72.7 ± 0.1; FL (FedSurF) AUC 73.6 ± 0.9. FL (FedSurF-IBS) AUC 73.7 ± 0.8
Späth et al. (26)	Time-to-event risk prediction	WHAS500	EHR	3 or 5 clients+ coordinator	FL Framework: FeatureCloud (federated Newton conjugate gradient) Core Model: Survival SVM (linear, regression objective) Strategy: secure aggregation via additive secret sharing	Cross-validation; Within-site; Centralized vs. FL; FL variants (with vs. without secure aggregation)	Harrell concordance index Centralized 0.76; FL (secure aggregation) 0.76; runtime increases with number of clients under secure aggregation
Yordanov et al. (27)	30-day mortality prediction after TAVI	Netherlands Heart Registration (NHR)	EHR	16 hospitals+server	FL Framework: FedAvg Core Model: LASSO-penalized logistic regression Strategy: central recalibration	Cross-validation+Held-out-site validation; Within-site+Cross-site; Local vs. Centralized vs. FL, FL variants	Local AUC 0.65 (95% CI: 0.63–0.67); Central AUC 0.68 (95% CI: 0.66–0.70); FL(FedAvg): AUC 0.67 (95% CI: 0.65–0.68);
Matsumoto et al. (28)	30-day mortality prediction in AMI	GUSTO-I trial	EHR	16 regions+server	FL Framework: FedAvg Core Model: Logistic regression Strategy: L1, L2, Elastic Net regularization	Cross-validation+Held-out-site validation; Within-site+Cross-site; Local vs. Centralized vs. FL	L1-regularized: Local AUC 0.733–0.846; Centralized AUC 0.779–0.847; FL (FedAvg) AUC 0.779–0.847
Zhou et al. (29)	30-day mortality risk prediction in PTE	Retrospective cohort from 12 hospitals (China)	EHR	5 hospitals+server; additional hospitals held out for testing	FL Framework: FedAvg Core Model: Logistic regression Strategy: feature standardization	Fixed split+Held-out-site validation; Cross-site; Local vs. Centralized vs. FL	Centralized AUC 0.811 ± 0.001 FedAvg (Real-world test set): AUC 0.835 ± 0.005
Heo et al. (30)	Critical intervention and poor outcome prediction at ED triage	Samsung Medical Center and Korea University ANAM Hospital ED cohort	EHR	2 hospitals+server	FL Framework: FedAvg Core Model: Shallow neural network Strategy: data-size–weighted aggregation	Internal split+Temporal validation; Within-site; Local vs. FL	Local model AUC 0.910 (95% CI: 0.905–0.915); FL (FedAvg) AUC 0.896 (95% CI: 0.891–0.902)
Bebortta et al. (31)	Heart disease hospitalization prediction	Boston Medical Center	EHR	5 clients+server	FL Framework: FedAvg Core Model: L1-regularized SVM Strategy: cluster primal–dual splitting (cPDS)	Internal split; Within-site; FL variants	Accuracy (1,000 rounds): Fed-SVM: 98.67% FL-SVM with cPDS: 99.86% Efficiency: Proposed method demonstrated significantly higher convergence rate and lower transmission cost.

Label definitions for validation design are the same as in Table 1.

AMI, acute myocardial infarction; AUC, area under the curve; CI, confidence interval; cPDS, cluster primal-dual splitting; ED, emergency department; EHR, electronic health record; FedSurF, federated survival forest; IBS, integrated Brier score; LASSO, least absolute shrinkage and selection operator; PTE, pulmonary thromboembolism; SVM, support vector machine; TAVI, transcatheter aortic valve implantation.

**Table 4:** Overview of FL applications in personalized medication and treatment of CVDs.

Ref	Clinical Task	Data source	Data Type	Client definition	Method	Validation design	Results
Kim et al. (32)	Personalized statin therapy recommendation	Korea University Hospital cohort	EHR	3 hospitals+server	FL Framework: FedAvg Core Model: Multi-layer perceptron Strategy: model selection by recall	Cross-validation; external validation; Cross-site; FL variants	AUC 0.85 (FL-MLP mean); Recall 0.76 (FL-MLP mean); FL-MLP selected for deployment due to the highest recall
Cadavid et al. (33)	Personalized incident CVD risk prediction	Lifelines Cohort Study	FHIR - tabular EHR	3 clients+server	FL Framework: FedAvg Core Model: Deep Cox proportional hazards network Strategy: FHIR-based data harmonization	Internal split; Cross-site; Local vs. FL (without aggregation)	C-statistic Global (without aggregation) 0.764; C-statistic Global (FedAvg) 0.788; monotonic improvement across aggregation iterations
Yurtoğlu et al. (34)	Personalized ECG-based cardiac classification	PTB Diagnostic ECG Database	ECG	3 sites+server	FL Framework: FedAvg (secure aggregation) Core Model: SVM Strategy: *χ*^2^ feature selection	Cross-validation; Within-site; Centralized vs. FL	FL: (SVM+χ^2^): Accuracy 0.85; F1-score 0.91; Centralized: (SVM+χ^2^) Accuracy 0.87; F1-score 0.93

Label definitions for validation design are the same as in Table 1.

AUC, area under the curve; C-statistic, concordance statistic; ECG, electrocardiogram; EHR, electronic health record; FHIR, Fast Healthcare Interoperability Resources; FL-MLP, federated learning multi-layer perceptron; MLP, multi-layer perceptron; SVM, support vector machine; χ^2^, chi-square feature selection.

We first recorded the clinical objective and the definition of the prediction target. This step helped us compare studies more clearly. We then recorded the data modality and key data-handling choices. These choices could affect cross-site validity and interpretability. We also described the federated setting, including the federation type, the multi-center configuration, the definition of clients, and, when available, the number of participating clients or sites, as shown in Tables 1–4. We then summarized the main methodological features, including the primary model or algorithm and, when applicable, any personalization strategy, as reflected in the “Method” field of Tables 1–4.

We separated the evaluation design from the comparator selection so that the reported performance could be interpreted consistently. We recorded whether studies used internal splits or cross-validation. We also noted whether studies reported temporal validation, held-out-site validation, or external validation, as summarized in the “Validation design” labels in Tables 1–4. We then recorded the main comparators. These comparators included local-only models, centralized training when allowed, and common federated baselines. We also extracted reported privacy and security mechanisms and deployment-related details. We summarized these aspects narratively because studies reported them inconsistently.

## 3 Methods

### 3.1 Search strategy

A comprehensive literature search was conducted to identify studies applying FL to CVD prediction and related clinical decision-support tasks. The primary database search was performed across PubMed, Web of Science, and IEEE Xplore, covering publications from January 2022 to December 2025. Supplementary searches were conducted in the arXiv e-print repository and Google Scholar, and reference lists of eligible articles were manually screened to identify additional relevant studies. Studies identified through these supplementary sources were included only if they met the same eligibility criteria as database-retrieved records.

Because FL-related terminology is inconsistently indexed across biomedical and engineering databases, MeSH terms were not used as the primary retrieval strategy. Instead, we used database-specific free-text strategies centered on the phrase “federated learning” in the title or abstract, combined with cardiovascular disease-related terms and clinical prediction terms. To maintain conceptual consistency and reproducibility across biomedical and engineering databases, we prespecified the review scope as studies explicitly identifying and implementing FL. Adjacent privacy-preserving distributed-learning approaches, including split learning, swarm learning, distributed ensemble learning, and other secure multi-institutional modeling methods, were outside the primary scope unless an FL framework was explicitly implemented.

A representative PubMed search string was [“federated learning” (Title/Abstract)] AND (“cardiovascular” OR “cardiovascular disease” OR “heart disease” OR “coronary artery disease” OR “heart failure” OR “myocardial infarction” OR “atrial fibrillation” OR arrhythmia OR cardiomyopathy OR “coronary heart disease” OR “pulmonary embolism”) AND (predict* OR “risk prediction” OR screening OR “early detection” OR diagnos* OR classification OR prognos* OR mortality OR readmission OR “treatment response” OR therapy OR personalized). Boolean operators AND and OR were used to refine retrieval. Similar search strategies were applied in Web of Science and IEEE Xplore with database-specific syntax adjustments. The final database search was completed in December 2025. Full search strings for all databases are provided in Supplementary Table S1.

### 3.2 Inclusion and exclusion criteria

**Figure 2:**
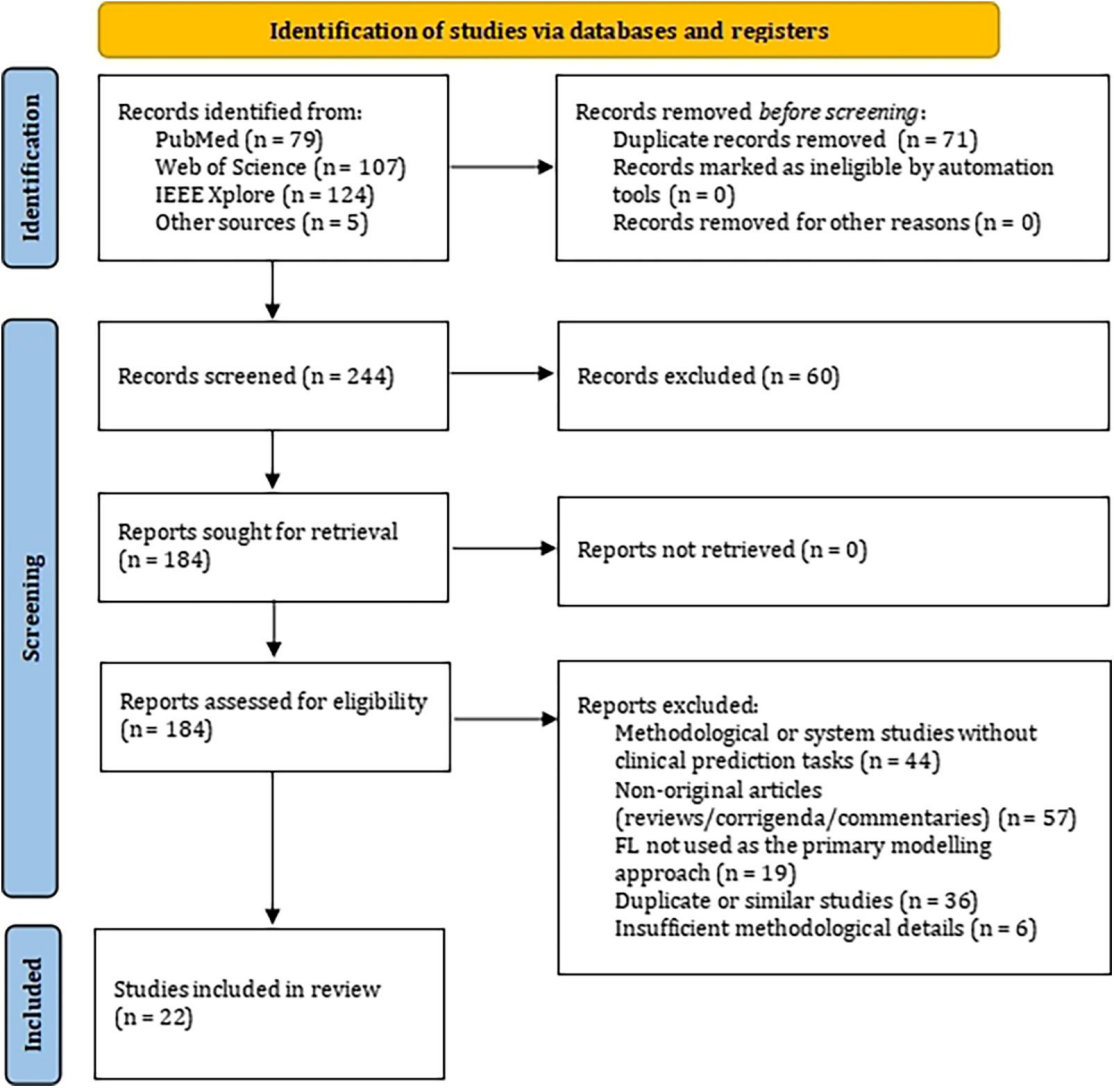
PRISMA flow diagram of the study selection process.

We imported all retrieved records into a reference management system. We removed duplicate records before screening. Two reviewers independently screened the titles and abstracts. The reviewers then assessed the full texts of potentially eligible studies. The reviewers resolved disagreements through discussion. A third reviewer was consulted when needed. Figure 2 shows the PRISMA flow diagram for study identification and selection.

We included original studies that explicitly implemented an FL framework and applied it to clinically meaningful cardiovascular decision-support tasks. For eligibility, FL was defined as collaborative model training across multiple clients or institutions in which patient-level data remained local and only model-related information, such as parameters, gradients, predictions, or intermediate updates, was exchanged. Studies using artificially partitioned public or centralized datasets were eligible only when they explicitly implemented an FL training procedure; however, these studies were classified as simulated federation during synthesis and were not interpreted as equivalent to real multi-site deployment. Eligible tasks included screening, diagnosis, risk prediction, prognosis evaluation, and treatment-related prediction in cardiovascular care. Eligible studies involved human cardiovascular populations or clinical datasets derived from cardiovascular care settings.

Peer-reviewed journal articles, conference papers, and preprints were eligible for inclusion, provided that sufficient methodological and evaluation details were available for interpretation. We excluded reviews, editorials, commentaries, non-cardiovascular applications, studies that did not explicitly implement FL, and reports with insufficient methodological detail for data extraction or interpretation.

### 3.3 Review methodology

This review was conducted as a systematic review. The report followed PRISMA 2020 guidance. After study selection, the eligible studies were examined using a structured qualitative synthesis. A meta-analysis was not performed because the included studies differed greatly in cardiovascular tasks, data modalities, FL formulations, validation strategies, and performance reporting. This heterogeneity made quantitative pooling inappropriate. The review aimed to summarize current applications of FL in cardiovascular prediction. It also aimed to highlight methodological trends, evaluation practices, and issues related to clinical translation.

### 3.4 Risk of bias assessment

Two reviewers independently assessed the risk of bias using the Prediction model Risk Of Bias Assessment Tool (PROBAST), which is designed for prediction model studies. PROBAST covers four domains: participants, predictors, outcome, and analysis. The reviewers resolved disagreements through discussion. A third reviewer was consulted when needed. Supplementary Table S2 summarizes the results of the risk-of-bias assessment.

### 3.5 Data extraction and synthesis

Each eligible study was charted using a standardized extraction form aligned with the analytical framework described in Section 2.2. Extracted items included the clinical task, data modality, federation setting, client construction, model or algorithm, validation design, comparators, performance metrics, privacy or security mechanisms, and deployment-related information. To support appraisal of translation readiness, federation settings were classified as real multi-site FL, simulated federation, or partially distributed federation. Real multi-site FL referred to studies using independent institutions or clinically distinct sites as clients; simulated federation referred to artificial client partitioning of centralized or public datasets; and partially distributed federation referred to multiple-dataset or pseudo-site settings that approximated real multi-site deployment.

Translation readiness was operationalized using the indicators summarized in Supplementary Table S3, including real multi-site participation, held-out-site or external validation, temporal validation, calibration, subgroup or fairness assessment, privacy and security mechanisms, system or communication metrics, and monitoring or update plans. These indicators were used as a structured appraisal framework rather than a formal scoring system. Validation terminology was also standardized: internal validation referred to random splits or cross-validation within development data; held-out-site validation referred to testing on sites excluded from training; external validation referred to testing in an independent cohort, institution, registry, or country; cross-dataset validation referred to testing across public datasets or databases; and temporal validation referred to testing on later calendar-period data. Greater interpretive weight was given to studies using real multi-site cohorts, stronger validation strategies, and more complete methodological reporting.

## 4 Applications of federated learning for cardiovascular disease prediction

Across the included studies, applications of FL for CVD prediction spanned the full cardiovascular care continuum, from early screening and diagnosis to prognosis evaluation and individualized treatment-related decision support. We first summarize the overall evidence profile across the included studies and then synthesize the evidence by clinical stage.

### 4.1 Overall evidence profile

The 22 included studies were distributed across four clinical categories: early screening (*n* = 4), clinical diagnosis (*n* = 8), prognosis evaluation (*n* = 7), and individualized medication or treatment-related prediction (*n* = 3). Diagnostic applications formed the largest group, whereas prognostic studies more often used registry, EHR, or hospital cohort data with clinically defined outcomes.

Translation-readiness indicators varied substantially across studies. Nine studies (40.9%) used real multi-site data, five (22.7%) used partially distributed or pseudo-site settings, and eight (36.4%) relied mainly on simulated federation. Held-out-site or external validation was reported in six studies (27.3%), and temporal validation was reported in one study (4.5%). Calibration was explicitly reported in two studies (9.1%) and partially reported in one additional study. Formal subgroup or fairness analyses were not explicitly reported in the included studies. Privacy or security mechanisms were clearly described in 11 studies (50.0%), mainly including differential-privacy-inspired perturbation, secure aggregation, encryption-based collaboration, secure multiparty computation, homomorphic encryption, or VFL-specific secure protocols. System or communication metrics were clearly reported in three studies (13.6%) and partially reported in nine additional studies. No study reported a complete post-deployment monitoring or model-update strategy.

**Figure 3:**
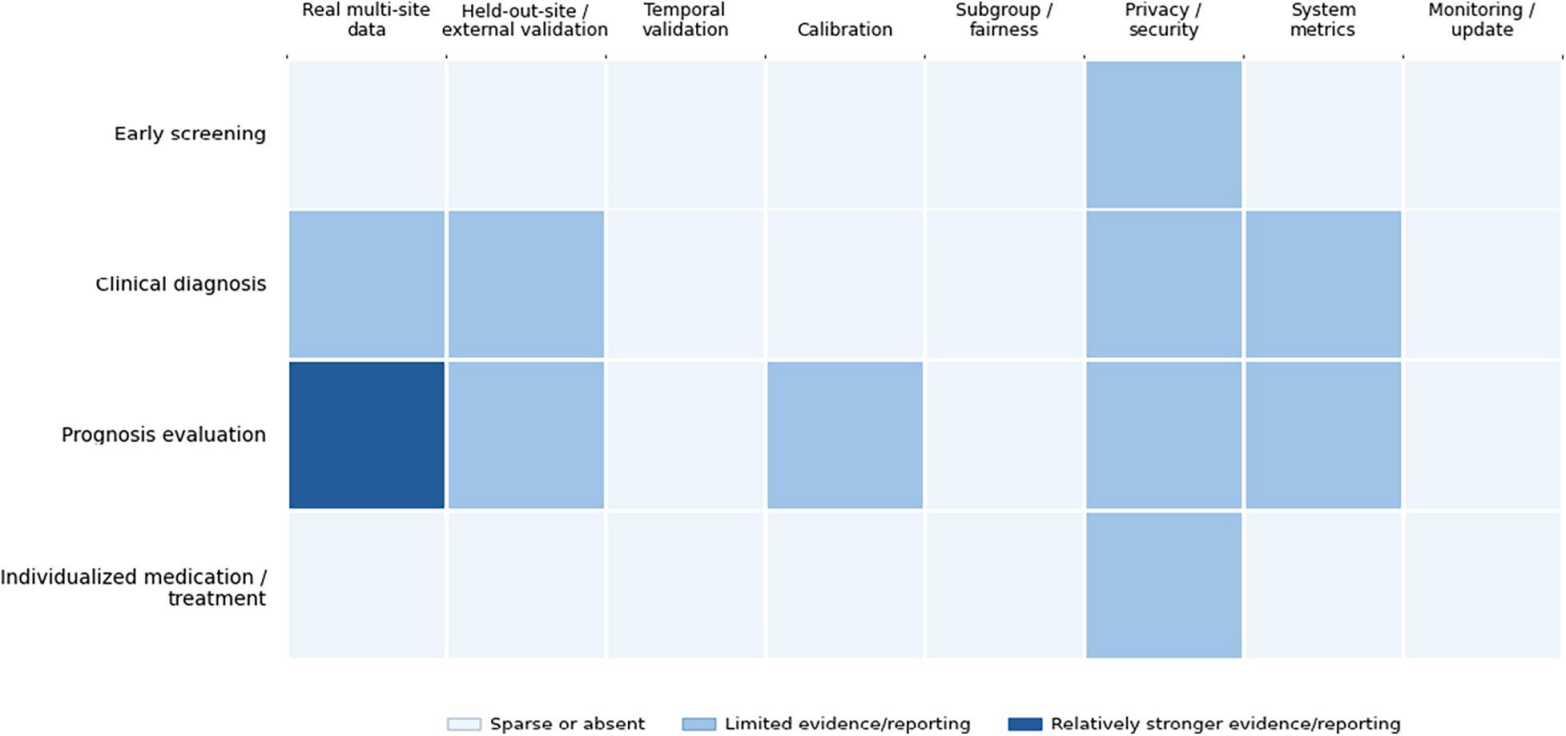
Evidence maturity landscape of federated learning studies for cardiovascular disease prediction.

These cross-study patterns provide context for the task-specific synthesis below. The evidence maturity landscape across clinical areas is summarized in Figure 3. The following subsections focus on the clinical task, data modality, federation design, validation strategy, and main translation-related limitations within each application area.

### 4.2 Early screening

Early screening in cardiovascular care aims to identify individuals at elevated risk or with early signs of disease, enabling timely confirmatory testing and preventive interventions. In the included literature, screening-stage applications of FL remain limited in number but already span heterogeneous modalities and objectives, including abnormal heart sound detection from phonocardiogram (PCG) signals, atrial fibrillation prediction from ECG signals, and multimodal early detection using combined clinical and physiological information. Key characteristics are summarized in Table 1.

**Figure 4:**
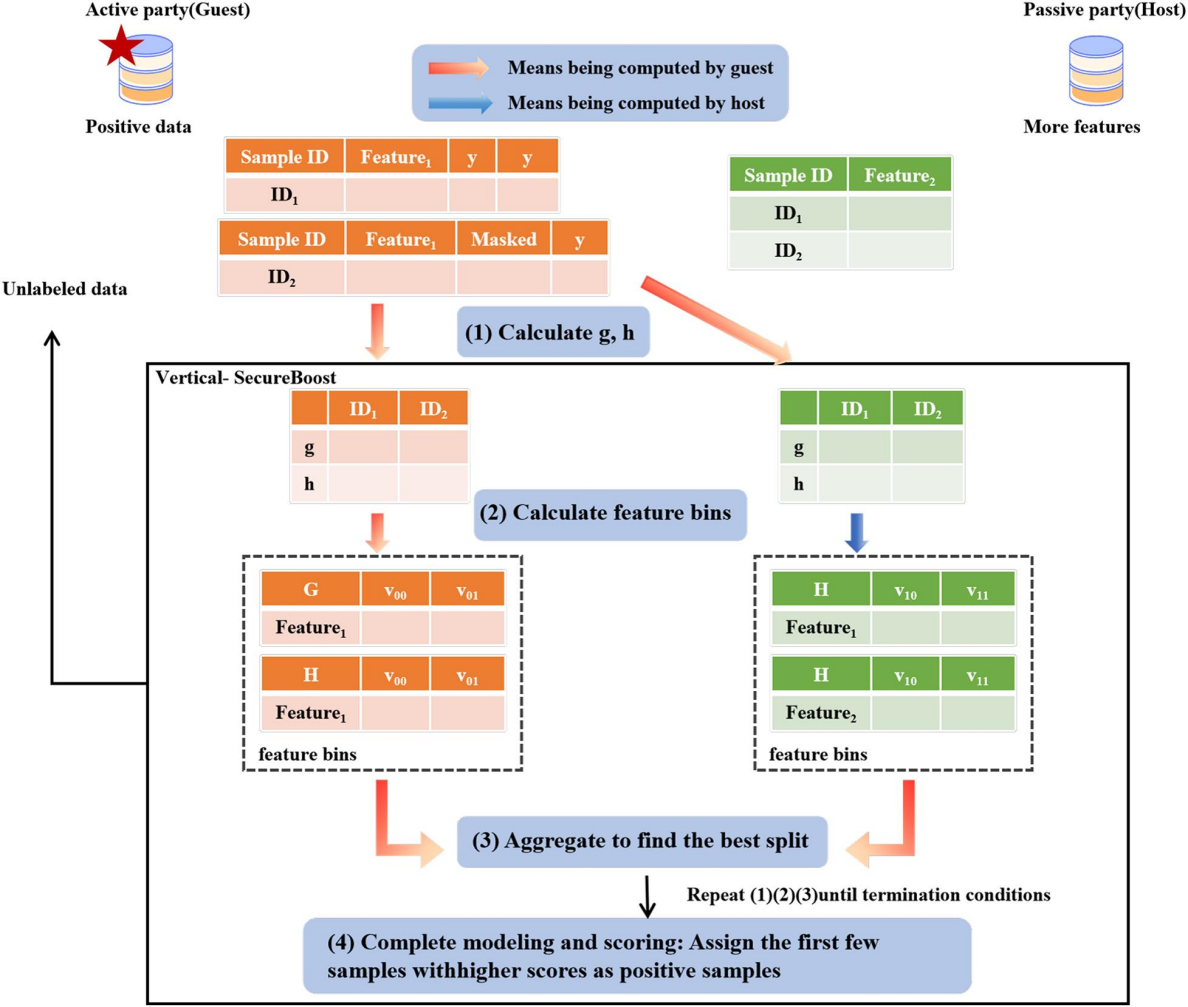
Overall workflow of the vertical secureBoost framework. Adapted from “Rough outline of the workflow of semisupervised vertical-SecureBoost” by Wanyong Qiu, Chen Quan, Yongzi Yu, Eda Kara, Kun Qian ,Bin Hu, Björn W. Schuller and Yoshiharu Yamamoto, licensed under CC BY 4.0.

Three of the four screening studies used horizontal FL or FedAvg-based variants as the main aggregation strategy (13, 14, 16), whereas one study used a VFL SecureBoost framework for feature-partitioned collaboration (15). Methodological emphasis varied according to the main screening challenge. The multimodal early-detection study used attention-based feature fusion and perturbed local updates before aggregation, reflecting a privacy-aware design within the screening workflow (13). One heart-sound classification study explored shared-data initialization to mitigate non-IID heterogeneity (14), while another heart-sound study combined vertical SecureBoost with positive-unlabeled learning to address weak or incomplete labels (15). The ECG-based atrial fibrillation study incorporated feature extraction and SMOTE-based oversampling to reduce class imbalance within a federated pipeline (16). The workflow of the vertical SecureBoost framework is summarized in Figure 4.

Screening-stage studies mainly demonstrated technical feasibility, but evidence for translation readiness remained limited. Three of the four studies relied on public datasets, simulated clients, or cross-dataset evaluation, and independent held-out-site or external validation was rarely reported. Calibration, subgroup performance, and operational feasibility were also rarely reported, restricting assessment of real-world robustness.

### 4.3 Clinical diagnosis

Clinical diagnosis and disease characterization require reliable phenotyping across institutions. Model development is still limited by governance requirements and site-specific heterogeneity. Among the eight diagnostic studies, four focused primarily on cardiovascular imaging tasks involving Echo, CT, or CMR (17–20), one combined ECG and Echo for multimodal HCM detection (21), and three used tabular clinical variables or benchmark heart-disease datasets for diagnosis or risk prediction (22–24). Imaging studies addressed automated measurement refinement, segmentation, landmark detection, and disease identification, whereas tabular-data studies focused more on diagnostic classification and training efficiency. Table 2 summarizes the study-level details.

Seven of the eight diagnostic studies used horizontal or shared-parameter federation with standard aggregation or aggregation variants (18–24), while one used sequential cross-site model transfer for echocardiographic measurement refinement (17). In imaging-based diagnosis, methodological contributions mainly targeted cross-site heterogeneity and annotation constraints, including heterogeneity-aware benchmarking for ECG and Echo tasks (18), semi-supervised federated knowledge distillation for cardiac CT (19), and equal-voting aggregation for CMR-based HCM diagnosis (20). The multimodal HCM study used ECG and Echo models in a stepwise screening framework across multinational cohorts (21). In tabular clinical-variable studies, the emphasis shifted toward feature selection, training stability, asynchronous updating, and optimization-driven aggregation (22–24). Benchmark evidence further suggests that center heterogeneity may coincide with long-tailed label distributions, amplifying performance variability across underrepresented cardiovascular phenotypes (18).

The diagnostic evidence was stronger when real multi-site cohorts and held-out-site or external validation were used. Two imaging studies reported held-out-site or leave-one-center-out validation (19, 20), and the multimodal HCM study included external validation in an independent cohort (21). However, other diagnostic studies relied mainly on internal splits, measurement-agreement evaluation, public datasets, or simulated client partitions. Calibration, deployment feasibility, and subgroup robustness were inconsistently reported, so high discrimination or segmentation performance should be interpreted together with validation design and client construction.

### 4.4 Prognosis evaluation

Prognostic evaluation in cardiovascular care focuses on short- to medium-term outcomes. These outcomes include mortality, clinical deterioration, and time-to-event endpoints. This type of evaluation often needs multi-center cohorts with consistent outcome definitions. Among the seven prognostic studies, five addressed short-term or clinical outcome prediction using registry, EHR, trial, or hospital-cohort data (27–31), whereas two focused on privacy-preserving time-to-event modeling using federated survival methods (25, 26). The reported applications included 30-day mortality prediction after TAVI or AMI (27, 28), prognosis assessment for acute pulmonary embolism (29), early risk stratification at ED triage (30), and heart-disease hospitalization prediction in an IoT-oriented EHR setting (31). Compared with screening and many diagnostic studies, prognostic applications more often use clinically defined outcomes and multi-institutional or registry-based data sources. One registry-based TAVI study included data from 16 hospitals and reported discrimination comparable to centralized training, but also showed calibration variability across external hospitals during geographic external validation (27). Table 3 summarizes the key characteristics of these studies.

Methodological choices in prognostic studies were closely linked to endpoint type. Three of the seven studies used logistic regression or penalized logistic regression for short-term mortality or prognosis endpoints (27–29), while two used survival-specific models, including federated survival forests and federated survival SVMs (25, 26). Other studies used shallow neural networks or SVM-based models for ED triage and EHR-based hospitalization prediction (30, 31). Several designs also incorporated federation-specific system or privacy considerations: additive secret sharing was used for privacy-preserving survival SVM aggregation (26), FedSurF++ reduced communication by constructing a global survival forest through selected local trees (25), and a Round-Robin-style client update schedule was used to reduce transmission cost in an IoT-oriented EHR setting (31).

Across prognostic applications, FL generally achieved discrimination close to centralized training and often outperformed local-only baselines, but the advantage varied across endpoints, sites, and validation designs. In ED triage, performance relative to local training varied across endpoints and centers under temporal validation (30). Cross-study interpretation is further constrained by heterogeneous validation protocols and inconsistent reporting of calibration. The TAVI registry study shows that acceptable discrimination may coexist with clinically consequential cross-site calibration variability during geographic external validation (27). Operational evidence remains comparatively sparse. When communication or convergence benefits are reported, they are often demonstrated in experimental or simulated settings, such as IoT-oriented implementations, rather than under deployment-grade evaluation (31).

### 4.5 Individualized medication and treatment

Treatment planning in CVD care is highly individualized. Regimen choice, behavioral intervention, and follow-up targets depend on patient-specific risk profiles, response patterns, and clinical constraints. In the included studies, treatment-oriented applications used FL to learn from distributed treatment and follow-up data. These models aimed to support personalized recommendations. Other studies embedded federated risk models into digital twin systems to support patient-specific monitoring and scenario exploration without sharing raw data.

The available studies can be grouped into two main design patterns. The first pattern links model outputs to a clear recommendation component. In this pattern, model outputs guide individualized decision rules or downstream intervention support, such as DRL-based lifestyle support modules (13). The second pattern defines personalization through treatment targets and goal attainment. In this setting, federated models estimate the probability of reaching a preset target after therapy begins. These estimates can be incorporated into decision-support interfaces for regimen selection (32). Another line of work places risk prediction in digital twin environments. This work focuses on patient-level monitoring and “what-if” exploration while keeping raw data local (33, 34). In these digital twin studies, reporting mainly focuses on feasibility and system-level behavior. These studies give less attention to calibration and clinical integration (33).

Overall, evidence for individualized medication and treatment optimization remains preliminary. Most demonstrations are retrospective or prototype-oriented, and reporting emphasizes discrimination or feasibility metrics with less consistent attention to calibration, subgroup robustness, workflow integration, and prospective evaluation. Descriptions of operational constraints and privacy assumptions also vary across studies, which limits between-study comparability and makes it difficult to judge translation readiness from performance metrics alone. Table 4 summarizes the studies in this subsection.

## 5 Discussion

FL has been studied across several cardiovascular prediction tasks, but the current evidence base remains uneven in maturity and completeness of reporting. Overall, the included studies support the technical feasibility of FL for CVD prediction, while evidence for deployment readiness remains more limited. Studies based on real multi-site data, clinically meaningful outcomes, and held-out-site or external validation provide stronger translation-relevant evidence, whereas studies relying mainly on public datasets, simulated clients, or internal validation are more difficult to interpret for real-world implementation. From a modality perspective, EHR- or registry-based prognostic modeling and selected cardiovascular imaging applications currently appear more mature for translation than PCG- or ECG-based screening studies, which more often rely on public datasets, simulated clients, or limited validation.

This hierarchy of evidence was consistent with the PROBAST assessment. Eighteen of the 22 studies were judged to have a high overall risk of bias, two had a low risk of bias, and two had an unclear risk of bias. High risk was driven mainly by the analysis domain, including limited calibration assessment, insufficient external or temporal validation, and incomplete reporting of model development procedures. Importantly, high reported accuracy, AUC, or F1-score did not necessarily indicate low bias or high translation readiness, particularly when studies used simulated clients, public datasets, or only internal validation. Therefore, discrimination metrics should be interpreted together with calibration, held-out-site validation, per-site robustness, and deployment-related reporting.

### 5.1 Heterogeneity and temporal drift

**Figure 5:**
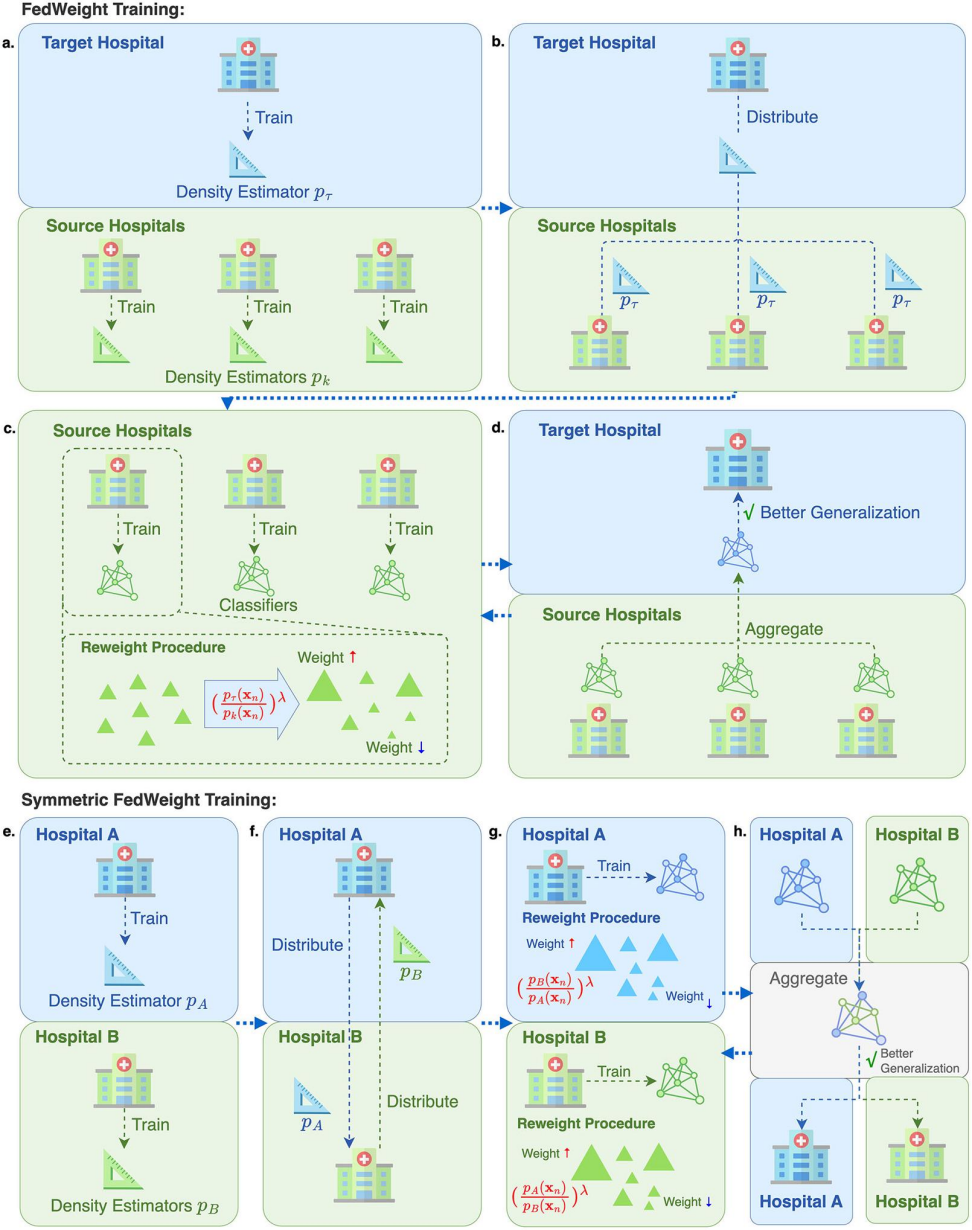
FedWeight training process. **(A)** In the asymmetric setting, the target hospital and source hospitals first fit their own density estimators locally. **(B)** The density estimator from the target hospital is then distributed to the source hospitals. **(C)** Based on the received estimator, each source hospital computes patient-level re-weighting factors and uses them during local model training. **(D)** The target hospital subsequently combines the source-hospital model parameters through the federated learning algorithm to improve generalization to the target population. In the symmetric FedWeight setting, **(E)** Hospitals A and B train density estimators independently, **(F)** exchange the learned density estimators, **(G)** treat the other hospital as the target site to estimate patient-level re-weighting factors for local training, and **(H)** aggregate the locally trained models through the federated learning algorithm. Reproduced from “FedWeight training process” by He Zhu, Jun Bai, Na Li, Xiaoxiao Li, Dianbo Liu, David L. Buckeridge and Yue Li, licensed under CC BY-NC-ND 4.0.

Clinical data distributed across hospitals and devices inherently violate the IID assumption. Variations in acquisition hardware, clinical pathways, patient demographics, and documentation practices introduce site-specific effects. These biases distort local training and can induce client drift, thereby compromising global convergence and cross-site generalization (35). In the context of CVD screening, federated heart sound studies have documented significant skew in both label distributions and sample quantities across clients (14). Similarly, multi-center benchmarks reveal that data heterogeneity frequently co-occurs with long-tailed label distributions, disproportionately affecting performance on underrepresented cardiovascular phenotypes (18). To address cross-hospital covariate shift in EHR-based prognostic modeling, patient-level reweighting, as implemented in FedWeight (36), has been proposed. The workflow of this approach is illustrated in Figure 5 (adapted from (36).

To support clinical translation, heterogeneity mitigation strategies can be grouped by their practical goals. Drift-reduction methods aim to stabilize aggregation in non-IID settings. These methods usually use constrained local updates or variance correction techniques (37, 38). Other methods focus on representation alignment and label-efficient pretraining. These methods aim to reduce cross-site feature differences, especially when labeled data are limited (39, 40). A key next step is careful comparison of these method families under consistent data partitions. Future studies should compare these method families under consistent data partitions and move beyond aggregate metrics by reporting per-site performance, held-out-site validation, calibration, and clinically meaningful subgroup analyses.

Personalization is often necessary because a single global CVD model rarely generalizes well across institutions, devices, or clinical workflows. Model decoupling and knowledge-transfer approaches allow a shared global backbone with local adaptation (41, 42), whereas meta-learning methods such as Per-FedAvg learn an initialization that supports rapid client-specific fine-tuning (43). Personalization targets should be defined clearly. Future studies should quantify the additional deployment burden introduced by personalization, including local fine-tuning, monitoring, and model-governance requirements.

Post-deployment heterogeneity introduces temporal drift. As cardiovascular guidelines, patient populations, and measurement protocols evolve over time, model updates risk inducing catastrophic forgetting (44, 45). Recent analyses suggest that client drift in space and catastrophic forgetting over time may come from the same source, which is a distribution shift. These problems should therefore be studied together (44). Evaluation protocols should combine held-out-site validation with calendar-time validation. Future studies should define update triggers that are linked to specific workflow changes. Drift monitoring systems should also support shadow evaluation, recalibration, or rollback when performance falls below predefined safety thresholds.

### 5.2 Privacy, security, and robustness

Privacy is often described as a main reason for using FL in CVD prediction. FL does not automatically guarantee confidentiality or integrity. Raw data stays local, but model updates can still leak sensitive information. Some participants may also send manipulated updates. These updates can weaken risk models used for triage or therapy planning (41). In multi-hospital CVD networks, the attack surface is broader because update traffic, model checkpoints, and system metadata move across institutional infrastructures. Any breach can damage trust and interrupt collaboration.

For clinical translation, protection strategies should be grouped by their practical roles. These strategies should not be treated as automatic features of federation. Secure aggregation and other cryptographic methods aim to prevent parties from seeing individual updates. These methods include secure multiparty computation and homomorphic encryption (46, 47). Reviews in healthcare indicate that cryptographic protection can entail high computational costs. These reviews also show that scalability remains a challenge in large multi-hospital deployments (48). Differential privacy aims to limit information leakage by adding calibrated noise. This method also requires a defined privacy budget across training rounds. In the included CVD studies, the cardioNet + framework used differential privacy by adding noise to client updates before aggregation (13). Related medical FL studies have also shown trade-offs between privacy protection and predictive performance under dual-mechanism designs (49). Robustness methods aim to protect model integrity when participants are adversarial or faulty. Work on these defenses is still less developed than work on heterogeneity and label quality in medical FL (41).

Trust should be treated as a reporting and evaluation standard. Studies should define clear threat models. Studies should also state which protection layers are used. Studies should report the added computational and communication costs. Privacy budgets should be reported in clear and understandable terms. Robustness claims should be tested under realistic attack and failure conditions. Deployed systems should also include key management, audit logging, and incident response procedures. These measures help maintain the credibility of privacy and integrity claims during repeated model updates and redeployments.

### 5.3 Operational feasibility and deployment evidence

Operational feasibility is a prerequisite for translating federated learning into cardiovascular disease prediction. In multi-site settings, communication overhead can dominate training time and cost. Among the included FL CVD studies, system reporting was limited, but some designs reduced transmission. FedSurF++ trained a multi-center survival model in a single communication round (25). FedEHR reduced communication overhead by rotating client participation with a Round-Robin schedule (31). Evidence from a 50-client testbed suggests that parameter communication can take more than half of the total training time (50). FedAvg is still a common baseline (51). In practical deployments, some methods reduce communication by using quantization and sparsification (52, 53). Other methods use system-level scheduling or client selection to reduce the effect of stragglers (54). Communication optimization methods, such as sparsification and selective synchronization, can also greatly reduce the transmitted volume (50, 55, 56). Many medical FL studies still do not report standard system metrics. This gap makes cross-study benchmarking difficult. At minimum, studies should report wall-clock time, transmitted volume, participation rates, dropout handling, and the overhead caused by security and privacy layers. These system-level items are incorporated into the broader reporting checklist proposed below.

Feasibility also depends on how well federated systems can fit into existing hospital informatics environments. Differences in EHR ecosystems, cybersecurity requirements, and local computing capacity make this difficult, and they require interoperable data mapping, consistent feature definitions, and harmonized labeling across institutions. For clinical translation, update pipelines should also be auditable and reversible, especially when models are revised repeatedly across sites. A technology readiness perspective further highlights the need for instrumentation, staged release, and rollback supported by logging and monitoring (57). Future studies should report integration assumptions and operational dependencies, including client resource budgets and update frequency, because these factors shape whether federated updates can be carried out safely without disrupting cardiovascular workflows.

Deployment evidence should also be linked to validation and monitoring. External validity should be assessed across time, place, and domain. The validation strategy should match the intended use. Geographic external validation assesses performance in independent locations, whereas held-out-site validation assesses generalization to sites excluded from model training. Temporal validation should use calendar-time splits that reflect changes in practice and patient populations (58). Post-deployment drift is hard to avoid, so monitoring is necessary after deployment. Current evidence on practical monitoring strategies for healthcare AI remains limited (59). Common drift proxies may also fail to consistently reflect clinically meaningful performance loss (59). Future FL-CVD studies should define update triggers, use shadow evaluation with clear rollback criteria, and report performance, calibration, and operational cost together in prospective multi-site evaluations.

### 5.4 Interpretability, transparency, and fairness

Interpretability and fairness are prerequisites for the clinical adoption of federated cardiovascular prediction, as clinicians must understand why a model issues a risk estimate and whether it behaves consistently across hospitals and patient subgroups. In current FL-CVD studies, interpretability is often assumed rather than explicitly evaluated, and formal assessment of explanation fidelity, cross-site stability, and fairness remains uncommon. This gap matters because federation limits centralized auditing and may obscure institution-specific drivers of cardiovascular risk. Naïvely aggregating feature importance across clients can reduce node-level fidelity and weaken interpretability at individual institutions (60).

For translation, interpretability should be framed as an explanation workflow rather than a catalogue of techniques. Local-first pipelines generate site-specific explanations and share only privacy-preserving summaries, then synthesize a global view using aggregation that reflects explanation uncertainty and accuracy (61). Model-intrinsic transparency, such as attention-based saliency, can provide intuitive cues. However, attention highlights associations rather than guaranteeing decision-critical evidence and may introduce additional computational overhead in federated settings (62). Federated knowledge distillation offers an alternative by transferring knowledge through shared predictions or intermediate representations, enabling student models to learn under privacy constraints without exchanging raw data (19). Fairness evaluation should consider both cross-site variation and subgroup disparities, since favorable global averages can conceal clinically relevant inequities.

These issues become more pronounced when federated cardiovascular prediction moves toward foundation and generative models. Foundation models and large language models may help leverage unstructured cardiology narratives for FL, which remain underrepresented in the current FL-CVD literature that has largely focused on structured EHR variables (25, 28, 29, 31, 36). These models impose higher trust, governance, and computational requirements, and may amplify site-specific documentation bias or generate unverified outputs (63). Parameter-efficient federated adaptation may reduce this burden by exchanging lightweight updates rather than full model parameters (64). Continual adaptation could help models track evolving cardiology terminology and practice patterns, but it also increases oversight requirements. Therefore, federated foundation and multimodal models require careful validation, uncertainty reporting, and bias auditing before clinical deployment.

Future work should use consistent evaluation standards, including per-site explanations, cross-site stability of explanations, clinically meaningful subgroup performance, and assessment under held-out-site or temporal validation. For foundation and generative models, evaluation should additionally address controllable generation, uncertainty reporting, institutional bias auditing, and deployment criteria that balance benefit, cost, and safety.

Taken together, the recurring limitation across heterogeneity, privacy, operational feasibility, interpretability, and fairness is incomplete reporting of information needed to judge reproducibility, robustness, and translation readiness. As a practical synthesis of these gaps, we propose a review-derived minimum reporting checklist for future FL-CVD studies (Table 5). This checklist complements existing prediction-model and clinical AI reporting guidance by emphasizing federation-specific items, including client construction, real vs. simulated federation, validation design, privacy and security assumptions, system metrics, and post-deployment monitoring.

**Table 5:** Minimum reporting checklist for FL-CVD prediction studies.

Domain	Minimum items to report
Clinical objective and intended use	Cardiovascular task, prediction target, prediction horizon, target population, intended clinical setting, decision point, and expected user.
Data source, governance, and client structure	Data modality, number of clients or sites, real multi-site vs. simulated or partially distributed federation, sample size and event count per client, inclusion and exclusion criteria, data-use or governance constraints when relevant, missing-data handling, label definition, feature harmonization, class imbalance, and non-IID characteristics.
Federation design and model development	HFL, VFL, or FTL setting; client definition and participation strategy; exchanged information; aggregation algorithm; model architecture; local epochs; number of communication rounds; batch size; optimizer; learning rate; personalization strategy; and hyperparameter tuning procedure.
Comparators and validation design	Local-only, centralized, and standard FL baselines where feasible; internal validation; cross-site testing; held-out-site validation; external validation; temporal validation; and clear separation between model development and testing data.
Performance and clinical evaluation	Discrimination metrics, calibration metrics, confidence intervals or uncertainty estimates, threshold-based metrics, per-site performance, performance variability across clients, subgroup or fairness analyses, error or failure-case analysis, and clinical utility assessment when applicable.
Privacy, security, and robustness	Threat model; privacy-preserving mechanisms, such as secure aggregation, encryption, SMPC, homomorphic encryption, or differential privacy; privacy budget when applicable; robustness to faulty, missing, or malicious clients; and computational or communication overhead caused by privacy or security layers.
System and communication metrics	Hardware and software environment, wall-clock training time, communication rounds, transmitted data volume, bandwidth assumptions, convergence behavior, client participation rate, client dropout, straggler handling, and scalability with increasing client numbers.
Deployment and monitoring	Data-pipeline integration, interoperability assumptions, update frequency, drift monitoring, recalibration triggers, shadow evaluation, rollback criteria, audit logging, and prospective or real-world evaluation plan when applicable.

## 6 Limitations of the review

This review has several limitations. First, the review followed PRISMA 2020 guidance but used narrative synthesis rather than a quantitative meta-analysis. This choice was made because FL studies differ widely across cardiovascular tasks, data modalities, multi-center settings, and reporting practices. Although appropriate for the heterogeneous current literature, the lack of quantitative pooling limits direct comparison of effect sizes across tasks, modalities, and validation designs. The synthesis, therefore, included a structured qualitative appraisal of evidence strength. This appraisal considered the study setting, the validation design, and the completeness of reporting.

Second, this review was designed to synthesize FL-specific evidence for cardiovascular prediction rather than all privacy-preserving distributed learning approaches. Requiring “federated learning” to appear in the title or abstract improved conceptual consistency and reproducibility across biomedical and engineering databases, but may have missed studies that used adjacent paradigms, such as split learning, swarm learning, distributed ensemble learning, or secure multi-institutional modeling, without explicitly describing the method as FL. Therefore, the findings mainly characterize the current evidence base for FL-CVD prediction and should not be generalized to all privacy-preserving learning paradigms in cardiology.

Third, the 2022–2025 review window captured recent developments but may still underrepresent earlier foundational work and very recent studies that were not yet indexed. Heterogeneity in endpoints, cohort definitions, client construction, validation protocols, and reported metrics also limited cross-study comparison.

Finally, the strength of inference is limited by the maturity of the evidence base. Many studies remain retrospective or rely on public datasets with simulated federation. Reporting of clinically relevant evaluation, operational feasibility, and privacy or system overhead is also inconsistent. Some real-world deployment constraints may therefore be underestimated because these details could only be extracted when studies reported them explicitly.

## 7 Conclusion

FL provides a practical paradigm for privacy-preserving, multi-institutional cardiovascular prediction by enabling joint model development without centralizing patient-level records. The reviewed applications span early screening, diagnosis, prognosis evaluation, and emerging individualized treatment support. However, the evidence base remains uneven: many evaluations are retrospective, validation protocols are heterogeneous, and screening-oriented studies often rely on public datasets or simulated client splits. Clinically aligned held-out-site and external validation, together with consistent reporting of calibration, subgroup performance, and operational feasibility, are therefore essential for translation.

Clinical translation will require methods that address non-IID heterogeneity, client drift, and personalization, supported by per-site reporting of calibration and fairness under realistic shifts. Scalable deployment also depends on communication- and system-efficient design, with bandwidth, wall-clock time, and participation patterns reported alongside predictive performance. Privacy, security, interpretability, and fairness should be treated as core requirements for high-stakes cardiology use. Emerging directions, including federated foundation models, LLMs, and drift-sensitive continual FL, may extend the field, but they require compute-efficient adaptation, deployment-grade monitoring, and clear governance.

## Data Availability

The original contributions presented in the study are included in the article/Supplementary Material, further inquiries can be directed to the corresponding author/s.
