# Supplementary material for: Federated learning for cardiovascular disease prediction: a systematic review of clinical applications, validation, and translation readiness
Source: Front Cardiovasc Med. 2026 Jun 8;13:1831342. doi: 10.3389/fcvm.2026.1831342 (PMC13284696; doi:10.3389/fcvm.2026.1831342)
Supplement: Supplementary file 1 [file Datasheet1.pdf]

Supplementary Material

1 Supplementary Tables

Table S1. Full search strategies used in each database for the systematic review

Database	Full search strategy
PubMed	(“federated learning” [Title/Abstract]) AND (“cardiovascular” OR “cardiovascular disease” OR “heart disease” OR “coronary artery disease” OR “heart failure” OR “myocardial infarction” OR “atrial fibrillation” OR arrhythmia OR cardiomyopathy OR “coronary heart disease” OR “pulmonary embolism”) AND (predict* OR “risk prediction” OR screening OR “early detection” OR diagnos* OR classification OR prognos* OR mortality OR readmission OR “treatment response” OR therapy OR personalized)
Web of Science	(TI=(“federated learning”) OR AB=(“federated learning”)) AND TS=(“cardiovascular” OR “cardiovascular disease” OR “heart disease” OR “coronary artery disease” OR “heart failure” OR “myocardial infarction” OR “atrial fibrillation” OR arrhythmia OR cardiomyopathy OR “coronary heart disease” OR “pulmonary embolism”) AND TS=(predict* OR “risk prediction” OR screening OR “early detection” OR diagnos* OR classification OR prognos* OR mortality OR readmission OR “treatment response” OR therapy OR personalized)
IEEE Xplore	((“Document Title”:“federated learning”) OR (“Abstract”:“federated learning”)) AND (“All Metadata”:“cardiovascular” OR “All Metadata”:“cardiovascular disease” OR “All Metadata”:“heart disease” OR “All Metadata”:“coronary artery disease” OR “All Metadata”:“heart failure” OR “All Metadata”:“myocardial infarction” OR “All Metadata”:“atrial fibrillation” OR “All Metadata”:arrhythmia OR “All Metadata”:cardiomyopathy OR “All Metadata”:“coronary heart disease” OR “All Metadata”:“pulmonary embolism”) AND (“All Metadata”:predict* OR “All Metadata”:“risk prediction” OR “All Metadata”:screening OR “All Metadata”:“early detection” OR “All Metadata”:diagnos* OR “All Metadata”:classification OR “All Metadata”:prognos* OR “All Metadata”:mortality OR “All Metadata”:readmission OR “All Metadata”:“treatment response” OR “All Metadata”:therapy OR “All Metadata”:personalized)

Table S2. PROBAST-based risk-of-bias assessment of included studies

Study	Participants	Predictors	Outcome	Analysis	Overall risk of bias
Alasmari et al. [13]	Unclear	Unclear	Unclear	High	High
Qiu et al. [14]	Low	Low	Low	High	High
Qiu et al. [15]	Low	Low	Low	High	High
Alreshidi et al. [16]	Unclear	Low	Unclear	High	High
Morbach et al. [17]	Low	Low	Low	High	High
Zhang et al. [18]	Low	Low	High	High	High
Tölle et al. [19]	Low	Low	Low	High	High
Linardos et al. [20]	Low	Low	Low	High	High
Goto et al. [21]	Low	Low	Low	High	High
Yaqoob et al. [22]	Low	Low	Low	High	High
Khan et al. [23]	Unclear	Low	Low	High	High
Houssein et al. [24]	Low	Unclear	Low	High	High
Archetti et al.[25]	Low	Low	Low	High	High
Späth et al.[26]	Low	Low	Low	High	High
Yordanov et al.[27]	Low	Low	Low	Low	Low
Matsumoto et al.[28]	Low	Low	Low	Low	Low
Zhou et al.[29]	Low	Low	Low	High	High
Heo et al.[30]	Low	Low	Low	Unclear	Unclear
Bebortta et al. [31]	Unclear	Unclear	Unclear	High	High
Kim et al. [32]	Low	Low	Low	Unclear	Unclear
Cadavid et al.[33]	Low	Low	Low	High	High
Yurtoğlu et al.[34]	Low	Low	Unclear	High	High

Table S3. Translation readiness indicators across included federated learning studies for cardiovascular prediction

Ref	Real multi-site	Held-out-site or external validation	Temporal validation	Calibration	Subgroup / fairness	Privacy / security	System metrics	Monitoring or update
Alasmari et al. [13]	No	No	No	No	No	Yes	No	No
Qiu et al. [14]	Partial	Partial	No	No	No	Partial	No	No
Qiu et al. [15]	Partial	Partial	No	No	No	Yes	No	No
Alreshidi et al. [16]	No	No	No	No	No	Partial	Partial	No
Morbach et al. [17]	Partial	No	No	No	No	Yes	Yes	No
Zhang et al. [18]	Yes	No	No	No	No	Yes	Partial	No
Tölle et al. [19]	Yes	Yes	No	No	No	Yes	Partial	No
Linardos et al. [20]	Partial	Partial	No	No	No	Partial	No	No
Goto et al. [21]	Yes	Yes	No	No	No	Yes	Partial	No
Yaqoob et al. [22]	No	No	No	No	No	Partial	Partial	No
Khan et al. [23]	No	No	No	No	No	Partial	Partial	No
Houssein et al. [24]	No	No	No	No	No	Partial	Partial	No
Archetti et al.[25]	Yes	No	No	Partial	No	Partial	Yes	No
Späth et al.[26]	Partial	No	No	No	No	Yes	Partial	No
Yordanov et al.[27]	Yes	Yes	No	Yes	No	Yes	No	No
Matsumoto et al.[28]	Yes	Yes	No	Yes	No	Yes	No	No
Zhou et al.[29]	Yes	Yes	No	No	No	Partial	No	No
Heo et al.[30]	Yes	No	Yes	No	No	Yes	No	No
Bebortta et al. [31]	No	No	No	No	No	Partial	Yes	No
Kim et al. [32]	Yes	Yes	No	No	No	Yes	No	No
Cadavid et al.[33]	No	No	No	No	No	Partial	No	No
Yurtoğlu et al.[34]	No	No	No	No	No	Partial	Partial	No

Note: Held-out-site or external validation was marked “Yes” when a study tested the model on a site, cohort, registry, country, or institution excluded from model development. Geographic external validation was considered a subtype of external validation. “Yes” indicates explicit reporting, “Partial” indicates incomplete reporting or approximate design, and “No” indicates absent or unclear reporting. Simulated federation refers to artificial partitioning of centralized or public datasets, whereas partially distributed federation refers to multiple datasets or pseudo-sites that approximate but do not fully reproduce real multi-site FL. Privacy/security was marked “Yes” when a specific mechanism, such as secure aggregation, encryption, SMPC, homomorphic encryption, differential privacy, update perturbation, or VFL-specific secure collaboration, was reported.
